# An Unusual Presentation of Large Glomangioma of the Hand

**DOI:** 10.7759/cureus.15936

**Published:** 2021-06-26

**Authors:** Abdullaziz K Alhujayri, Sultan Alshehri, Ziyad Alghweinem, Obaid Almeshal

**Affiliations:** 1 Plastic Surgery Division, Department of Surgery, Ministry of National Guard - Health Affairs, Riyadh, SAU; 2 King Abdullah International Medical Research Center, Ministry of National Guard - Health Affairs, Riyadh, SAU; 3 College of Medicine, King Saud bin Abdulaziz University for Health Sciences, Riyadh, SAU

**Keywords:** glomangioma, hand tumors, solid tumors, perivascular tumor, hand mass

## Abstract

Glomangiomas are rare and benign hamartomas that commonly occur in the upper extremities. It is not typical for benign glomangiomas to be larger than one centimeter in size, and they usually present as a faint, blue-red subungual papule associated with a triad of symptoms of paroxysmal pain, pain with cold exposure, and tenderness to touch.

We herein report a case of a 72-year-old man with multiple comorbidities presented to our clinic as a case of right-hand middle finger swelling for the past five years. Initially, it was not painful. However, the pain became more noticeable when he lowers his hand, and it was relieved when he kept it elevated. There were no skin changes around it with minimal tenderness over the swelling. Hand MRI demonstrated a well-defined small lobulated nodule at the radial aspect of the middle finger, at the level of the middle phalanx with no invasion to an adjacent structure. Surgical excision was done and the patient was diagnosed by histopathology to have glomangioma.

Glomangiomas, also known as glomus tumors, are rare and benign hamartomas that commonly occur in the upper extremities. The hand is the most common site for glomus tumors, particularly the subungual area, the lateral aspect of the digits, and the palms. Female patients are the most common to present with subungual glomangioma. Multiple papers reported different presentations, and due to the rarity of the conditions and overlapping in clinical and imaging characteristics with other conditions, it was challenging to diagnose.

Such atypical cases must be approached with high clinical suspicion and proper imaging and investigations so as to not delay diagnosis and management.

## Introduction

Glomangiomas are rare and benign hamartomas that commonly occur in the upper extremities [1]. Glomangiomas are usually under one centimeter in size and present as a faint, blue-red subungual papule associated with a triad of symptoms of paroxysmal pain, pain with cold exposure, and tenderness to touch. Histologically, they consist of glomus bodies [2]. Glomangiomas are rarely found to be malignant. Those that are malignant tend to be over two centimeters and found intravenously [3]. The most common site is the hand, particularly the subungual area, the lateral aspect of the digits, and the palms [4]. On MRI, glomangiomas typically show high-signal intensity on T2-weighted images and avid enhancement following IV gadolinium administration [5]. We present a case of a 72-year-old man who presented with a five-year history of right-hand middle finger swelling and recent progressive dull middle finger pain that was suspicious and required urgent intervention.

## Case presentation

**Figure 1:**
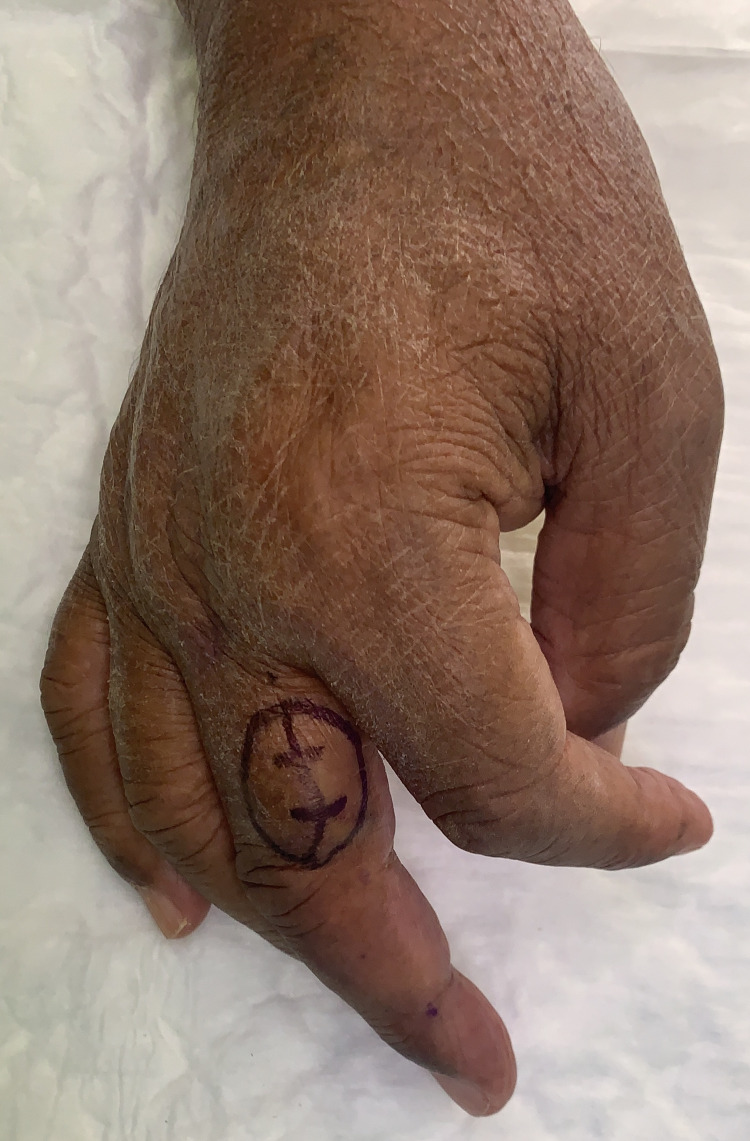
The mass marked before the surgical excision. The marking was done in a mid axial fashion to access the mass directly and evaluate the neuromuscular bundle due to its proximity to it.

A 72-year-old man with multiple comorbidities presented to our clinic as a case of right-hand middle finger swelling for the past five years. It started to bother him in the last five months. Initially, it was not painful. However, the pain became more noticeable when he lowers his hand, and it was relieved when he kept it elevated. On examination, there was a soft swelling on the dorsal aspect of the radial side of the right middle finger middle phalanx about 3 cm x 2 cm in size. There were no skin changes around it with minimal tenderness over the swelling. He has a full range of motion with intact sensation (Figure 1).

**Figure 2:**
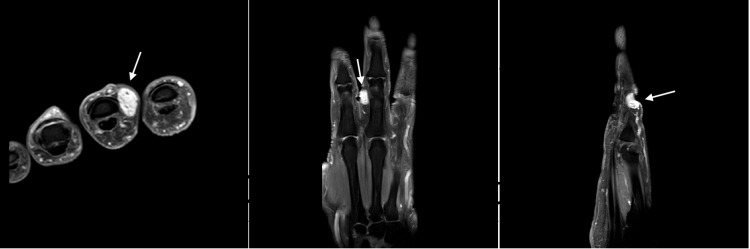
MRI T1 axial (left), coronal (middle), and sagittal (right) sections of the hand showing hyperintense lesion of the radial aspect of the middle finger. The arrows on the different cuts show the location of the mass on the middle finger.

Hand X-ray did not show any abnormalities. Hand MRI demonstrated a well-defined small lobulated nodule at the radial aspect of the middle finger, at the level of the middle phalanx. This nodule is measuring about 1.2 cm x 0.7 cm in anteroposterior (AP) and transverse diameter, respectively, with no invasion to adjacent structure (Figure 2).

**Figure 3:**
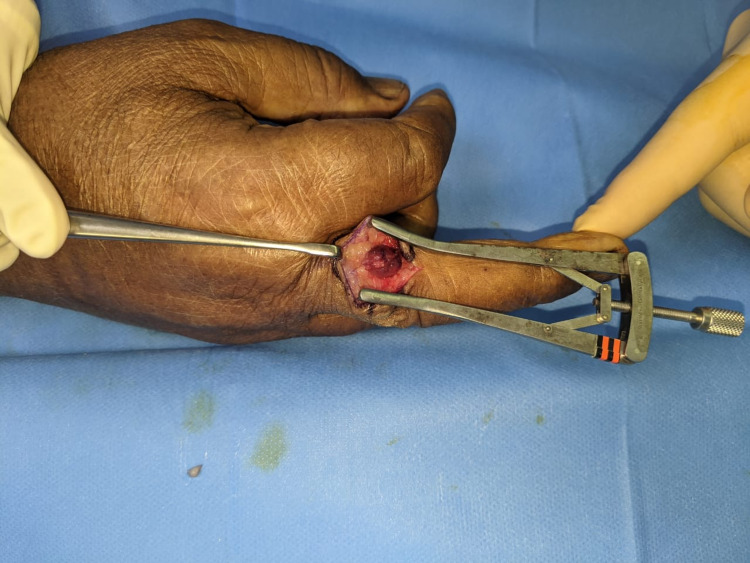
A picture of the lesion during the excision after it has been freed from the surrounding structures before excision.

The patient was then booked for an excisional biopsy of the swelling. It was a well-loculated mass with a feeding vessel without any attachments to the surrounding structures (Figure 3). Pathology gross description demonstrated a round piece of tan-brown soft tissue measuring 1.2 cm x 0.9 cm x 0.5 cm. Bisecting reveals a heterogenous tan-gray cut surface. Microscopic description demonstrates a well-circumscribed mass with a prominent vasculature component containing dilated capillary-sized vessels lined by endothelial cells surrounded by collars of uniform glomus cells with indistinct borders in a hyalinized stroma. A diagnosis of glomangioma was made.

## Discussion

Glomus tumors refer to hyperplasia of the glomus body. A glomus body is an apparatus of the skin and a type of arteriovenous anastomosis that controls the body temperature. Glomangioma found in the subungual area mostly occurs in female patients [6] and it accounts for 1%-5% of soft tissue tumors of the hand [2]. Glomangiomas present as a faint, blue-red subungual papule associated with a triad of symptoms of paroxysmal pain, pain with cold exposure, and tenderness to touch [2]. In 1812, Wood first described a glomus tumor as a painful subcutaneous nodule made worse by changes in temperature and treated by surgical removal [7].

 Histologically, they consist of glomus bodies, smooth muscles, and blood vessels. Glomus tumors can be identified as three types depending on the composition of the tumor: glomangiomas mainly consist of blood vessels; solid glomus tumors are mainly composed of glomus cells, and glomangiomas mainly consist of smooth muscles. While glomus tumors are essentially benign, glomus tumors in rare cases are accompanied by sarcomas to form glomangiosarcoma [8].

 A variety of common and uncommon soft tissue tumors often have overlapping imaging characteristics, the appropriate diagnosis of which can be a source of confusion for both clinicians and radiologists [9]. On MRI, glomangiomas typically show high-signal intensity on T2-weighted images and avid enhancement following IV gadolinium administration [5]. They are typically hypoechoic on ultrasound with internal Doppler signal and can be painful with direct pressure by the ultrasound probe [10].

 The symptoms and clinical presentation of paroxysmal pain, tenderness, and aggravation of the symptoms by coldness are significant in establishing a diagnosis. A study done by Lee et al. retrospectively reviewed 15 patients with subungual glomus tumors of the hand. All of them were presented with a chief complaint of pain in a subcutaneous nodule in the hand. In all of the presentations, the pain was caused by pressure, but in one case, the pain occurred as cold intolerance. Three cases featured nail deformities. Seven showed bluish discoloration in the nails. All of the patients had a solitary glomus tumor in one digit [4].

 Bordianu and Zamfirescu published a report of a patient with a glomus tumor on the dorsolateral side of the distal phalanx of the left thumb associated with intermittent episodes of severe pain and cold intolerance [1]. Another study done by Mravic et al. reviewing 138 cases over a period of 14 years elucidated clinicopathologic correlation in terms of clinical, radiological, histopathological, and immunohistochemical features of these lesions. Illustrating trends in the clinical versus pathologic diagnoses of glomus tumor, 99 of the cases were diagnosed clinically, while 137 of the cases were diagnosed by histopathology. Of the 99 clinical diagnosis cases, only 45, just under half, were diagnosed by pathological examination. While of the 137 cases that were diagnosed by histopathology, only 45 were clinically diagnosed based on the clinical picture. Only 18.2% of the cases employed the use of imaging, none of the imaging studies used suggested a diagnosis of glomus tumor. Furthermore, physicians in the study were more likely to consider glomus tumor as the sole clinical diagnosis if the lesion was present in a classic location on the fingers or fingertips. However, if the lesion presented elsewhere, a clinical differential was more often listed. Within the study, tumors were most commonly found in the digits, followed by the arm and hands, then legs, trunk, and less commonly the deep-seated tumors in the gastric, duodenal, and subglottic locations [11]. This could possibly show that the relative rarity of glomus tumors and its overlapping features with other similar soft tissue tumors of the hand make it a difficult diagnosis to establish.

 In comparison to previous studies, our patient presented with a benign three-centimeter glomangioma of the hand for five years that only recently became painful when lowering the hand. The patient did not mention cold intolerance. And there were no skin changes, with minimal tenderness over the swelling. Giving an initially unclear clinical picture and recent changes of the symptoms, it raised the suspicion of malignant changes. The use of imaging was needed. Hand X-ray did not show any abnormalities. Hand MRI only demonstrated a well-defined small lobulated nodule at the site of the mass. Surgical excision was done and the patient was diagnosed by histopathology with having glomangioma.

## Conclusions

Despite the well-documented clinical features of glomus tumor and that glomus tumors and glomangiomas are a well-known cause of chronic pain in the digits, atypical clinical presentations of glomangioma that are not associated with paroxysmal pain, tenderness, and cold intolerance could obscure the clinical picture thus potentially delaying diagnosis and treatment. As physicians, we should keep an open mind and have high suspicion when we face such cases with atypical presentations that indicate further investigation and prompt intervention.
